# Implementing Fixed Dose Combination Medications for the Prevention and Control of Cardiovascular Diseases

**DOI:** 10.5334/gh.860

**Published:** 2020-08-19

**Authors:** Ruth Webster, Adrianna Murphy, Helen Bygrave, Éimhín Ansbro, Diederick E. Grobbee, Pablo Perel

**Affiliations:** 1 Centre for Health Economics Research and Evaluation, University of Technology Sydney, AU; 2 The George Institute for Global Health, The University of New South Wales, Sydney, AU; 3 Centre for Global Chronic Diseases, London School of Hygiene and Tropical Medicine, London, UK; 4 Médecins Sans Frontières Access Campaign, Geneva, CH; 5 UMC Utrecht, Utrecht, NL; 6 World Heart Federation, Geneva, CH

**Keywords:** Cardiovascular Prevention, Polypills, Hypertension, Public Health, Combination therapy

## Abstract

**Highlights:**

- Despite clinical evidence of its effectiveness in secondary prevention of cardiovascular disease, uptake of fixed dose combination therapy (FDCs) for CVD has been poor.
- A symposium was held bringing together stakeholders on this issue, including from academia, government and NGOs.
- The conclusion made was that what is now needed to improve implementation of FDCs is country-specific health systems analyses to design appropriate implementation strategies.
- Implementation strategies must look beyond listing on the WHO Essential Medicines List to consider approaches to improving FDC availability, accessibility, affordability, and adherence.
- Strategies might include incorporation of FDCs into the WHO HEARTS technical package, simplified treatment and monitoring algorithms, decentralisation of medicine dispensing and task-sharing for treatment management.

## The Problem

The political declaration on non-communicable diseases (NCD) adopted by the UN general assembly in 2011 acknowledged the growing epidemic of NCDs that is occurring primarily in low and middle-income countries (LMIC). In response to this, the World Health Organization (WHO) global action plan (25 × 25 goals) outlined nine global voluntary targets to reduce premature death from the four major NCDs by 25% by 2025 [1]. Included in these is the target to have at least 50% of eligible people (defined as age 40 years or older with a 10-year calculated cardiovascular risk of at least 30% or established cardiovascular disease) receiving drug therapy and counseling to prevent heart attack and stroke by 2025. An associated target is to achieve 80% availability of affordable, safe, and efficacious essential medicines for the management of NCDs, including cardiovascular disease. The more recent Sustainable Development Goals are aligned with the WHO 25 × 25 plan and aim to reduce premature NCDs by one third by 2030.

There is high-quality evidence of efficacious and safe medicines to reduce the risk of further cardiovascular events for people with established CVD (including statin, blood pressure-lowering drugs, and aspirin) and those diagnosed with hypertension. However, these medicines are currently largely not taken by the majority of high-risk patients globally. The reasons for this are complex, including interwoven factors at the health system level, prescriber, and patient levels. These include poor availability of drugs, lack of affordability and/or access for the patient, lack of affordability to the health system, the complexity of multi-pill regimens for the patient, number of pills required to be prescribed (associated with physician inertia) and poor adherence by the patient. In the context of CVD preventive medications, the PURE study, a large cross-sectional study conducted in 18 countries, showed that the majority of communities in LMIC had less than 60% availability of recommended CVD preventive medications and that availability could be as low as 3% [2]. Affordability of these medications was also shown to be limited, with the proportion of households at risk of being unable to afford essential drugs ranging from 20% in the highest income earners to 88% in the lowest income earners. Unsurprisingly, proportions of people with established CVD in these communities who take all recommended CVD preventive medications are extremely low [3]. Even in high-income settings, where availability and affordability are generally less of a problem than in low-income settings, adherence rates (proportion of patients who take their medications as prescribed) are consistently low and average around 50% [4] or even lower [5,6]. Innovative strategies involving improved medication availability, affordability, access, and prescribing and adherence support to improve the use of CVD preventive medications globally are required if the 25 × 25 target is to be achieved. The commonality of risk factors responsible for CVD between high-income countries and low-income countries ensures that any solutions found, may have the potential (within the bounds of adaptation to the local context) to provide benefits for a broad range of countries across the economic spectrum.

## Precedent for Using Fixed Dose Combinations (FDCs) to Transform Access to Care

There are well-established precedents for the wide use of fixed-dose combination medications (FDCs) for chronic illnesses. For example, FDCs are recommended as first-line treatment in clinical guidelines for tuberculosis [7] and HIV [8]. With HIV specifically, development of a one pill, once a day, minimal toxicity regimen that could be applied across subpopulations, has allowed decentralization of treatment and task-sharing by simplifying prescribing and monitoring requirements [9]. Available research on this approach to HIV treatment has shown improved adherence to therapy [10], as well as patient and provider preference over multi-pill regimes [11]. The potential for operational benefits of FDCs has also been noted by experts convened by WHO at a roundtable in 2003 who reported increased security of supply systems and lower drug supply costs, associated with improved production, storage, transport, dispensing and other health system components [9]. Treatment guidelines incorporating these simplified regimens have thus moved away from an individualised approach and taken a public health approach, identifying the best option for the majority and ensuring that they have continued access whilst recognising that alternatives are needed for the minority that may have medical contraindications and/or side effects. Availability of FDCs and this public health approach to guidelines are acknowledged as key components in the successful strategy to scale-up HIV treatment globally [9]. The benefits of FDCs for CVD prevention and control are likely to be similar.

## Evidence for FDCs in CVD Prevention and Control

The combination of multiple classes of low-cost generic CVD preventive medications into one pill was initially proposed by WHO in 2001 [12]. It was noted in this report that FDC medications had the capacity to overcome poor adherence to treatment (due to a multiplicity of pills); inadequate dosage (due to variability in prescribing practices of physicians); and cost and availability barriers of drugs. FDCs, therefore, have the capacity to address multiple points in the chain of potential barriers to adequate CVD preventive and treatment medication coverage.

### FDC for secondary prevention

Several randomized, placebo-controlled trials have now confirmed that FDCs improve blood pressure and cholesterol compared to usual care [13], and to the same degree and with similar rates of adverse events compared to individual component drugs [14,15,16,17]. Most recently, the first FDC trial to look at CVD outcomes (PolyIran) found a reduction in major CVD events among the polypill group compared to minimal care (adjusted hazard ratio (HR) 0.66, 95% CI 0.55–0.80) and this reduction was more pronounced when analysing those with the highest adherence alone (adjusted HR 0.43, 95% CI 0.33–0.55) [18].

Four trials (UMPIRE, IMPACT, Kanyini-GAP, and FOCUS) have assessed the effects of FDCs in patients either with an established disease or at high calculated risk (≥15% over five years) on adherence, LDL cholesterol, and systolic blood pressure compared to either usual care or separate drug components [19,20,21,22]. All four trials have shown improvements in adherence to CVD preventive medication with the use of a polypill with absolute increases ranging from 25% to 40% at 12 months. A meta-analysis of the Single Pill to Avert Cardiovascular Event (SPACE) Collaboration group of 3 trials (UMPIRE, IMPACT, and Kanyini-GAP) also showed an improvement in SBP (–2.46 mmHg, 95%CI: –4.55 to –0.37) and LDL-C (–0.09 mmol/L, 95%CI: –0.18 to 0.00) [23].

In the SPACE Collaboration trials, the trial design did not require stabilisation of patients on polypill component drugs at specific doses prior to randomisation. In fact, the largest benefits (RR 4.46, 95% CI 3.72 to 5.36) were seen in patients who were not receiving all recommended treatment at baseline. No safety issues were identified with this approach, thereby supporting the use of FDC therapy not only as a substitution therapy for patients already stabilised on existing medications but also as ‘step-up’ therapy to immediately move patients from being on no treatment or partial treatment to full treatment.

Analysis of reported safety data in trials to date shows no safety concerns as yet. However, broader post-marketing surveillance is recommended [24].

### FDCs for hypertension

The evidence for benefit in FDCs for the treatment of hypertension is well established, showing clear improvements in adherence [25] as well as the reduction in cardiovascular events for patients using combination therapy vs. individual tablets [26]. Additionally, the recent TRIUMPH study [27] a randomised controlled trial of low dose triple combination therapy vs. usual care demonstrated a significant improvement in the proportion of patients reaching their blood pressure targets within six weeks when a low dose triple combination pill was prescribed for patients either not on therapy or uncontrolled on monotherapy, thus supporting the early use of low dose combination therapy within a simplified treatment regimen to improve early blood pressure control. Guidelines such as the 2017 American Heart Association Hypertension guidelines [28] already endorse the use of FDCs in hypertension to improve adherence, with the initiation of treatment with multiple drugs recommended in patients with blood pressure > 20/10 mmHg above their target. The 2018 European Society of Hypertension guidelines have also moved to broaden their recommendations to acknowledge that combination therapy is now recognized as the most effective initial treatment strategy in most patients, with no specific threshold for initiation of combination therapy [29].

## Potential Global Impact of FDCs for CVD

Making an affordable FDC available in low- and middle-income countries has, therefore, the potential to contribute significantly to the global WHO CVD goals in a cost-effective manner and will assist in meeting the goal for coverage of preventive medications in at least 50% of the population [1,30]. Additionally, use of FDCs is fully compatible with established WHO strategies for CVD prevention as multidrug therapy is already recognised as a ‘best-buy’ by WHO [31], and recommendations for the use of FDCs is included in WHO recommendations including the WHO HEARTS technical package for management of cardiovascular disease which includes two suggested regimens for blood pressure control incorporating use of combination therapy [32].

## Scaling up FDCs for CVD

Successful scale-up of FDCs in low- and middle-income countries will require listing on the WHO Essential Medicines List (EML) as WHO endorsement is viewed as highly important, if not essential, by partner countries for any proposed scale-up or implementation of specific drug therapy.

Three applications (with increasing amounts of evidence) have been made to the EML since 2013 for the inclusion of CVD polypills onto the list, with all three applications being rejected. Several reasons for rejection have been reported by the WHO EML expert committees, including the perceived complexity of management of hypertension and cardiovascular disease, the need to individualise dose through titration of medications, and the challenge to prioritise which of the multiple available FDCs should be included. The differences with FDC use for infectious diseases (which are indeed included in the WHO EML and have been successfully scaled up) have also been stated. The need for FDCs to avoid microbial resistance is, of course, recognised as a feature exclusive to infectious disease. However, the benefits noted above, both for the health system and for patient adherence, should provide valuable programmatic experience to support their use in scale-up activities.

Progress has been seen, however, with the successful listing of four different combinations of FDCs for hypertension on the WHO EML in 2019 (Lisinopril/amlodipine, Lisinopril/hydrochlorothiazide, telmisartan/amlodipine, telmisartan/hydrochlorothiazide).

To address these challenges with obtaining EML approval for FDCs, and with the objective to bring together different perspectives and stakeholder views about this matter, a symposium was organized on July 3^rd^ by the London School of Hygiene & Tropical Medicine, Centre for Global Chronic Conditions, The George Institute for Global Health, and Médecins Sans Frontières. The symposium brought together cardiovascular and infectious disease research communities, governments, WHO, funders, private sector, and civil society, among others.

One of the main conclusions of this symposium was that simply making CVD FDCs (both polypills and FDCs for hypertension) available on the WHO EML and in the market is unlikely to have a large effect by itself. In order for CVD FDCs to be scaled up, they should be included as part of a larger strategy (as has been done for HIV, for example) for improving availability, accessibility, affordability, and adherence to medicines, and the design of appropriate health system delivery models [33]. Among other components, this strategy should consider the implementation of FDCs as part of the broader WHO HEARTS [32] technical package. Other components of the strategy should consider how to decentralise and task-share care through simplified treatment and monitoring algorithms such as those utilised in the treatment and management of HIV/AIDS. The rapid expansion of digital technologies, including the development of electronic decision support tools, should be harnessed to support FDC implementation. Use of such technology can facilitate prompt, time-efficient implementation of simplified FDC treatment algorithms and support community-driven diagnosis and management. Development of clinical guidelines on the use of FDCs, endorsed by national opinion leaders, Ministries of Health, and CVD organisations, could also play an important role in integrating these formulations into national public health-based programmes. Importantly, any implementation strategies must be designed with a pragmatic approach, evaluated in real-world settings, and refined accordingly. A follow-up meeting of this symposium, including WHO and other key stakeholders, including researchers and funders, is planned to take concrete steps towards developing a global strategy supporting the implementation of FDCs for CVD.

## Summary

Achieving the WHO 25 by 25 targets of at least 50% of eligible people receiving drug therapy and counselling to prevent heart attack and stroke and achieving 80% availability of essential medicines requires novel approaches. Use of single medicines from various drug classes, rather than combinations of such medicines, to address the risk associated with established CVD thus far has failed to provide a scalable, global solution to address the growing epidemic of CVD. FDC therapy in patients at high risk of CVD events has been shown to be effective in improving adherence and coverage of essential preventive medications and could be a core component to achieve WHO goals if part of global CVD prevention and control strategy.
